# The seventh survey of the Tromsø Study (Tromsø7) 2015–2016: study design, data collection, attendance, and prevalence of risk factors and disease in a multipurpose population-based health survey

**DOI:** 10.1177/14034948221092294

**Published:** 2022-05-04

**Authors:** Laila A. Hopstock, Sameline Grimsgaard, Heidi Johansen, Kristin Kanstad, Tom Wilsgaard, Anne Elise Eggen

**Affiliations:** 1 Department of Community Medicine, UiT The Arctic University of Norway, Tromsø, Norway; 2 Northern Norway Regional Health Authority, Tromsø, Norway

**Keywords:** Population studies, epidemiology, cohort studies

## Abstract

**Aims:**

The Tromsø Study is an ongoing population-based health study in Tromsø, Norway, initiated in 1974. The purpose of the seventh survey (Tromsø7) 2015–2016 was to advance the population risk factor surveillance and to collect new types of data. We present the study design, data collection, attendance, and prevalence of risk factors and disease.

**Methods:**

All inhabitants in Tromsø municipality, Norway, aged 40 years and older (*N*=32,591) were invited to a health screening including extensive questionnaires, face-to-face interviews, biological sampling (blood, urine, saliva, nasal/throat swabs, faeces), measurements (anthropometry, blood pressure, pulse, pulse oximetry) and clinical examinations (pain sensitivity, echocardiography, cognitive, physical, and lung function, accelerometer measurements, eye examinations, carotid ultrasound, electrocardiography, dual-energy X-ray absorptiometry, and heart, lung and carotid auscultation). New research areas in this round were dental and oral health examinations, collection of faecal samples for studies of normal bacterial flora and antibiotic resistance, and 24-hour urine samples for examination of sodium and iodine intakes.

**Results:**

Attendance was 65% (*N*=21,083), and was higher in women, age group 50–79 years, previous attenders, and Norwegian-born individuals. Cardiovascular risk factor levels and prevalence of chronic obstructive lung disease decreased since the last survey, while the prevalence of obesity and diabetes increased.

**Conclusions:**

Attendance was stable from the sixth survey. Interaction with participants might be key to maintain participation. Favourable trends in risk factors continue, except for a continued increase in obesity. Both new data collection technology and traditional physical examinations will be crucial for the impact of future population studies.

## Background

### Tromsø Study

The Tromsø Study [1] is an ongoing population-based health study anchored at UiT The Arctic University of Norway (UiT). The study started out as a combined cardiovascular risk screening and research study, in which the overall aim was to combat the cardiovascular disease epidemic in the 1970s [2]. Currently, the Tromsø Study is a multipurpose health survey including both chronic and communicable diseases and risk factors, with a wide range of possible epidemiological and clinical research designs including registry linkage. Both complete birth cohorts and random samples of the population in Tromsø, Norway, have been invited to seven repeated surveys: Tromsø1 (1974, attendance 74%, *N*=6595, age 20–49 years); Tromsø2 (1979–1980, 74%, *N*=16,621, 20–54 years); Tromsø3 (1986–1987, 75%, *N*=21,826, 12–64 years); Tromsø4 (1994–1995, 77%, *N*=27,158, 25–97 years); Tromsø5 (2001, 79%, *N*=8130, 30–89 years); Tromsø6 (2007–2008, 66%, *N*=12,984, 30–87 years) and Tromsø7 (2015–2016, 65%, *N*=21,083, 40–99 years) (altogether *N*=45,473 attended one or more surveys). Data collection includes questionnaires and interviews, biological sampling, and clinical examinations. Tromsø4 and onwards consisted of a first visit (total sample) and a consecutive second visit (subsample) with additional clinical examinations. The purpose of Tromsø7 was to advance the long-standing population risk factor and disease surveillance, and collect new types of data.

## Aims

We will present the design, data collection, attendance, and sample characteristics including the prevalence of risk factors and disease.

## Methods

### Population

Tromsø is the largest municipality in northern Norway (2015: 73,000 inhabitants) with both urban (80%) and rural living areas. The population is mainly of Norwegian origin (85%). Residents are mainly employed in tertiary (trade, health service, education, public administration) and a lesser proportion in secondary and primary industry.

### Design

Most previous research areas were expanded in scope with regard to the amount of data collected, including extension of mental health and trauma data, and new questions about sexual health and illegal drug use. New research areas were clinical examinations of dental and oral health, collection of faecal samples for studies of normal bacterial flora and antibiotic resistance, and 24-hour urine samples for measurements of sodium and iodine intake. Questionnaires and examination protocols were aligned with the Russian Know Your Heart study [3] for comparison of population health in Norway and Russia as the ‘Heart to Heart’ initiative. Several Tromsø7 subprojects have innovation potential, including the use of AI technology. Machine learning techniques applied to raw data from, for example, accelerometers [4] and digitised electrocardiograms [5] can be used to predict health outcomes. Recordings of lung and heart sounds have been used to develop a teaching library for health personnel [6].

### User involvement

Users of the Tromsø Study include the municipality, the county, health authorities, healthcare providers, participants, and the general public. Users were involved on a strategic level, and in the planning of subprojects. The Tromsø7 steering group included representatives from the Norwegian Institute of Public Health, Northern Norway Regional Health Authority, Troms County, Tromsø municipality, University Hospital of North Norway (UNN), and the Norwegian Health Association. Users, including healthcare providers and representatives from patient organisations, were also involved in the detailed planning of subprojects and piloting of questionnaires.

### Sample

All inhabitants aged 40 years and older (*N*=32,591) were invited to visit1. A subsample (*N*=13,028) was pre-marked to extended examinations approximately 3–4 weeks later to visit2, consisting of a random sample of 20% aged 40–59 years and 50% aged 60–84 years (*n*=9925) as well as previous participants attending dual-energy X-ray absorptiometry (DXA), echocardiography and/or eye examinations in Tromsø6 (*n*=3103). Invitation to visit2 required attendance at visit1.

### Communication for recruitment and dissemination of results

We developed a communication strategy plan to enhance attendance, communicate preliminary results, and acquire funding. We recruited participants as study ambassadors, and researchers to present their research. Continuous outreach activities were conducted throughout the study period. Men in the younger age groups were specifically targeted, as they were less likely to attend. Target groups for funding and support were local, regional, and national stakeholders, health authorities, and funding bodies. We developed extensive profiling material for use in various communication channels including social and traditional media, and meetings with the public, patient organisations, non-profit organisations, as well as public and private enterprises.

### Ethics and privacy

Tromsø7 was approved by the Regional Committee of Medical and Health Research Ethics North (reference 2014/940) and the Norwegian Data Protection Authority (reference 14/01463-4/CGN). Participants signed consent forms at attendance. Data were stored in EUTRO, a UiT-developed IT system to collect, store and retrieve sensitive data and integrated biobank and project information, evaluated and approved by the Norwegian Data Protection Authority.

### Data collection

We piloted data collection prior to study start. This included a separate pilot for completion and content of questionnaires in older adults.

Invitations for visit1 were sent by mail weekly to randomly selected residents from 4 March 2015 to 26 June 2016. Non-attenders received two reminders (first: weeks 36, 47, 48; 2015; and 9, 10, 25, 26, 31, 32, 35; 2016; second: weeks 33, 34, 36, 37, 38; 2016).

The invitation included an information brochure and a personal letter with user name and password for completion of three online questionnaires: the short general Q1; long general Q2; and graphical index of pain (GRIP) questionnaire. Q1 was also included as a 4-page paper questionnaire to complete on paper or online. The information brochure included information about data collection, test result feedback, privacy, data storage, consent, and use of data including linkage to medical records and registries.

Study site location was close to a large shopping centre with easy access to parking and public transport. The first participants attended on 11 March 2015, the last on 29 October 2016. Examinations took place 6 days/week Mondays–Wednesdays 08:30–15:30, Thursdays 12:00–18:00, Fridays 08.30–14:00, and Saturdays 10:00–14:00 hours. Visit1 appointment time was given in the invitation letter, opting drop-in. Visit2 invitation was provided at visit1, as a fixed time booking Mondays–Fridays 08:00–16:00 hours.

**Figure 1.**
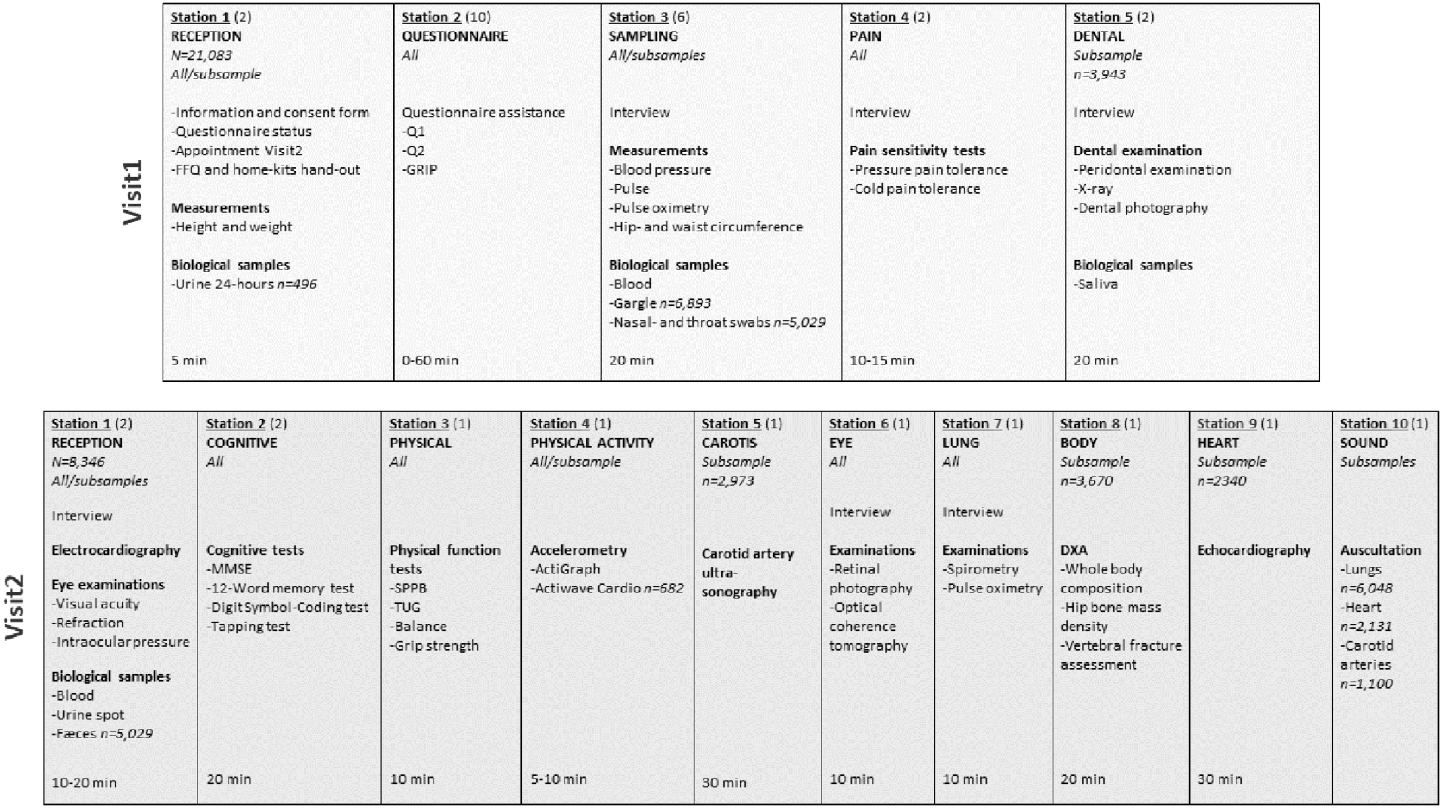
Data collection logistics. The Tromsø Study 2015–2016. Each column represents an at-site examination station with listed measurements, consecutively numbered as they appeared for the participants. The first row includes visit1 examinations, second row visit2 examinations. Numbers in parentheses are the number of stations for each examination. The number of participants are included for examinations or analyses in subsamples only. %"FFQ: food frequency questionnaire; GRIP: graphical index of pain; Q1: short general questionnaire; Q2: long general questionnaire; MMSE: mini-mental state examination; SPPB: short physical performance battery; TUG: timed up and go test; DXA: dual-energy X-ray absorptiometry.

Examination logistics is shown in Figure 1. Visit1 reception included signing of consent form, check of online submission of Q1 (if paper: check of completion) and Q2, measurement of height and weight, hand-out of the food frequency questionnaire (FFQ) and home kit for urine and faecal samples. Alternatives for return of FFQ and home samples was to reception (FFQ, 24-hour and spot urine), at visit2 (morning spot urine, faeces), or by mail (FFQ, faeces). If non-submitted Q1 (if paper: completion) and/or Q2, participants completed the questionnaires on available tablets/PCs prior to other examinations. Research technicians provided technical support if impairments, digital illiteracy, or need of translation (English only). At visit2 reception, electrocardiography, visual acuity, refraction, and intraocular pressure tests and blood sampling were performed, prior to other examinations. Specially trained research technicians performed all examinations. Adverse events related to data collection were registered throughout the study period.

### Questionnaires and interviews

Questionnaire Q1 and Q2 [7] covered social demographics, mental and somatic health, symptoms and disease, use of medication and healthcare services, lifestyle, wellbeing, quality of life and travels. This included validated instruments and diagnostic tools; single items, full or modified versions.

The GRIP questionnaire [8] is a pain screening instrument with a hierarchical body map of body regions and pain loci to give detailed information about pain location, distribution, and characteristics.

A validated FFQ [9] on paper was handed out to all participants at visit1, to be completed at site or returned by mail. The FFQ captures the full diet, and includes 261 questions on food, dishes, dietary supplements, meals, and beverages, to calculate energy, food, and nutrient intakes.

All questionnaires were in Norwegian, and full versions of Q1, Q2 and FFQ (including English translation of Q1 and Q2) are available at the Tromsø Study webpage [7]. From the pilot studies, the estimated mean (range) time for completion of online questionnaires was for Q1 13 (10–17) and Q2 55 (35–74) minutes. At-site interviews were related to biological sampling (e.g. time since last meal, pregnancy) or to clinical examinations (e.g. medication use, symptoms).

### Biological samples

Blood and urine samples were analysed within 24 hours at UNN (laboratory ISO certification NS-EN ISO 15189:2012) (Supplemental Table I). Additional samples were frozen, for the biobank and for later analysis of environmental pollutants, DNA, RNA, etc.

### Biological samples visit1

Non-fasting blood samples were obtained with a light tourniquet released after venepuncture. Serum samples were processed after 30–60 minutes in room temperature, centrifuged for 10 minutes at 2000***g***, transferred within 1 hour to plastic tubes, kept between 1°C and 10°C. Twenty-four hour urine with spot urine samples [10] were collected in a random subsample (*n*=496, 82% of invited) for analysis of sodium and iodine intake. Nasal and throat swabs were collected consecutively from March 2016 (*n*=5029) using sterile cotton-tipped swabs transferred to plastic tubes and frozen for later metagenomic analysis. Gargle samples were collected in a random subsample (*n*=6893), centrifuged for 15 minutes at 450***g*** and frozen for later analysis of human papillomavirus. Saliva samples from all attending dental examination (*n*=3880) were collected in plastic tubes (Oragene DNA self-collection kits, DNA Gentotek Inc.).

### Biological samples visit2

Non-fasting blood samples were obtained as in visit1. Morning spot urine was collected for three consecutive days. Faecal samples [11] were collected using a self-sampling kit including a nylon-flocked swab (ESwab 490CE.A; Copan, Brescia, Italy) (*n*=5042, 87% of invited).

### Clinical examinations visit1

Weight and height were measured with light clothing and no shoes with a Jenix DS-102 scale (DongSahn Jenix, Seoul, Korea). Waist and hip circumference were measured with a Seca measurement tape at the level of the umbilicus and the greater trochanters, respectively. Pulse and blood pressure were measured on the right arm three times at one-minute intervals after 2 minutes seated rest with a Dinamap ProCare 300 (GE Healthcare, Norway). Pulse oximetry was conducted with an Onyx II 9550 pulse oximeter (Nonin Medical, Inc., Plymouth, MN, USA) three times after 15 minutes of rest, of which the highest value was used.

Pressure pain tolerance was measured with computerised cuff algometry (NociTech, Aalborg, Denmark) with cuffs on both legs. The cuff was inflated by 1 kilopascal/second to the maximum pressure the participant could endure (maximum 100 kilopascal). Cold pain tolerance was measured by the cold pressor test, with the dominant hand submerged into a container with 3°C water, as long as the participant could endure (maximum 120 seconds).

Dental and oral health examinations were performed in a random subsample (*n*=3943) and included periodontal probing depth (PPD), bleeding on probing (BOP), one orthopantomogram (OPG) (Planmeca ProMax S3 2D DIMAX), four bite wing X-rays (BWs), and eight close-up clinical intra-oral digital photos of all teeth (Canon EOS 60D, Canon 105 mm; Sigma EM-140 DG). PPD was measured with a periodontal probe (WHO probe LM555B). BOP was measured in conjunction with the periodontal probing. PPD and BOP were assessed at four sites per tooth for all teeth. Dental caries, fillings and erosions were based on examination of BWs and clinical intra-oral photos. Caries were assessed as described by Mulic et al. [12] and dental erosions as by Mulic et al. [13]. OPGs were used to assess radiographic marginal bone level (MBL) and root filled teeth. The MBL of both distal and mesial surfaces of all teeth, excluding third molars, were measured linearly with a transparent plastic ruler on the OPG as described by Holde et al. [14] and Schei et al. [15].

### Clinical examinations visit2

Twelve-lead resting electrocardiograms were recorded with a computer-based electrocardiograph (Cardiovit AT-104 PC; Schiller AG, Baar, Switzerland).

Cognitive function was assessed as: (a) a global score of long-term verbal memory, episodic memory, and the ability to use learning strategies with the mini mental state examination [16]; (b) short-term verbal memory with a 12-word memory test [17]; (c) perceptual processing, speed and memory with the Wechsler adult intelligence scale digit symbol-coding test [18]; and (d) psychomotor speed with the tapping test [19].

Physical function was assessed using the original protocols with: (a) short physical performance battery including a timed walk test, chair sit-to-stand test, and balance tests (side-by-side, semi-tandem, and full tandem) [20]; (b) timed up and go test [21]; (c) one-legged balance with eyes open and eyes closed [22]; and (d) grip strength following the Southampton protocol [23] using a Jamar+ Digital Dynamometer (Patterson Medical, Warrenville, IL, USA) in which the participants received verbal encouragement from the tester to squeeze as hard as they could.

Physical activity [24] was measured with an ActiGraph wGT3X-BT accelerometer (ActiGraph, LLC, Pensacola, USA) worn 8 full days and nights on the right hip with an elastic band (*n*=6333, 93% of invited). A subsample additionally wore an Actiwave Cardio accelerometer with a one-channel ECG monitor (CamNtech, Cambridge, UK) for one full day and night (n=699). Accelerometers were returned by mail.

High-resolution B-mode ultrasonography of both carotid arteries was performed with a GE Vingmed Vivid 7 duplex scanner (GE Vingmed Ultrasound AS, Horten, Norway) with a linear 12 MHz transducer. Plaques were assessed in the far and near walls of the distal common carotid, the bifurcation, and the proximal internal carotid arteries. Automated R-triggered measurements of intimamedia thickness were performed in the far and near walls of the distal common carotid and in the far wall of the carotid bifurcation.

Eye examinations included: (a) visual acuity and refraction measured with a Nidek ARK 560a autorefractor (Nidek Co., Ltd., Japan); (b) intra-ocular pressure with I-care tonometer (model TA01i; Helsinki, Finland) on both eyes; (c) retinal photography with a Visucam 500 (Carl Zeiss Meditec, Jena, Germany); and (d) optical coherence tomography of both eyes (512 × 128 macular cube and optic disc cube protocol) with a Cirrus HD-OCT 4000 (Carl Zeiss Meditec, USA).

Spirometry was performed using SensorMedics Vmax 20c Encore (VIASYS Healthcare Respiratory Technologies, Yorba Linda, CA, USA), with daily calibrations, following the standards of the American Thoracic Society/European Respiratory Society [25], with no discontinuation of medication. Pulse oximetry was conducted with an Onyx II 9550 pulse oximeter (Nonin Medical, Inc., Plymouth, MN, USA) after 15 minutes of rest.

Body composition (fat and lean mass), bone composition (bone mineral content, area, and density) and vertebral deformities were measured using the GE Lunar Prodigy DXA (GE Healthcare, Norway) with daily calibrations. Participants underwent whole body, hip, and lateral scans. Post-scan images were inspected and corrected.

Echocardiography [26] was performed with a GE Vivid E9 ultrasound scanner (GE Medical, Horten, Norway). Offline image reading was performed using EchoPac software (EchoPac version 113; GE Medical, Horten, Norway).

Heart, lung and carotid artery sounds [27] were recorded with a Sennheiser microphone placed in a Littmann Classic II stethoscope tube.

Intra and interobserver studies were performed for pain sensitivity, physical function, carotid ultrasound, DXA, and echocardiography. Grip strength was measured in a subsample using the Martin Vigorimeter (KLS Martin Group, Tuttlingen, Germany) in addition to the Jamar dynamometer. A subsample wore the AX3 (Axivity, Newcastle, UK) accelerometer used in HUNT4 [28] in addition to ActiGraph and Actiwave Cardio.

### Feedback to participants

The feedback system for deviant test results (predefined cut-off values and type of response) was developed in collaboration with clinical specialists (Supplemental Table II). The response included immediate action (at-side or telephone contact) or letters. All participants were sent a letter 2–4 weeks after attendance including blood pressure, height, weight, body mass index, total and high-density lipoprotein cholesterol, glycated haemoglobin (HbA1c), and haemoglobin levels. Participants exceeding reference levels were advised to contact their general practitioner. Participants with deviant test results from other analyses and examinations were sent additional letters with specific recommendations. Cut-off for referrals from clinical examinations were similar to Tromsø6 [29]. In addition, participants with dental caries/bone loss/advanced PPD were advised to contact a dentist, participants with low D vitamin levels were invited to hospital follow-up/clinical trial participation, and participants wearing ActiGraphs were sent a summary letter of their activity level and sedentary time.

## Results

**Figure 2.**
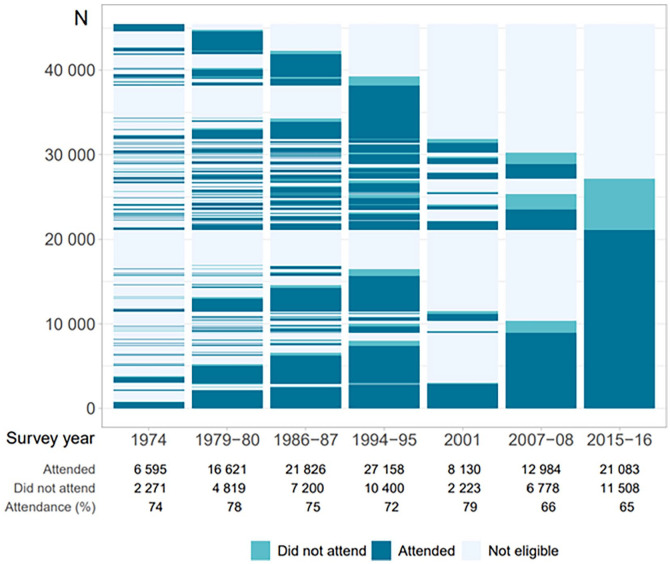
Lasagna plot of all participants Tromsø1 to Tromsø7, sorted by Tromsø7 attendance. The Tromsø Study 2015–2016. ‘Did not attend’ is defined as did not attend among all invited (figure text) or attended other surveys and was invited but did not attend the present survey (figure bars).

A total of 21,083 women and men aged 40–99 years attended visit1 (65%). Of these, 9253 were pre-marked for visit2 invitation. In total 8346 attended visit2 (comprising 64% of the originally pre-marked visit2 sample, 90% of those attending visit1). The number of participants for each examination is shown in Figure 1. Return of Q1, Q2, GRIP and FFQ were 99.9%, 98.9%, 96.1% and 71.8%, respectively, with high completion, also for potentially sensitive questions. Numbers of participants for each analysis of biological samples consecutively performed during data collection are shown in Supplemental Table I. Participant attendance is shown for visit1 and visit2 (Tables I and II), visit1 according to first-time invitees and previous attenders (Supplemental Table III), country of birth (Supplemental Table IV) and visit2 random and non-random samples (Supplemental Table V). Attendance was higher in women, age group 50–79 years, previous attenders, and Norwegian-born individuals. A lasagna plot gives an overview of participation in Tromsø1 to Tromsø7 sorted by participation in Tromsø7 (Figure 2).

**Table I.** Attendance at visit1 according to sex and age: the Tromsø Study 2015–2016.

Age, years	Women			Men			Total		
	Invited	Attended	%	Invited	Attended	%	Invited	Attended	%
40–49	5195	3378	65.0	5562	3054	54.9	10,757	6432	59.8
50–59	4534	3245	71.6	4327	2790	64.5	8861	6035	68.1
60–69	3586	2677	74.7	3543	2502	70.6	7129	5179	72.6
70–79	2001	1361	68.0	1897	1315	69.3	3898	2676	68.7
80–89	981	389	39.7	639	325	50.9	1620	714	44.1
90–104	242	24	9.9	84	23	27.4	326	47	14.4
Total	16,539	11,074	67.0	16,052	10,009	62.4	32,591	21,083	64.7

Values are numbers and proportions.

**Table II.** Attendance at visit2 according to sex and age: the Tromsø Study 2015–2016.

Age, years	Women				Men				Total			
	Eligible^a^	Invited^b^	Attended	%^c^	Eligible^a^	Invited^b^	Attended	%^c^	Eligible^a^	Invited^b^	Attended	%^c^
40–49	1071	713	641	89.9	1117	618	540	87.4	2188	1331	1181	88.7
50–59	1186	891	816	91.6	1006	683	603	88.3	2192	1574	1419	90.2
60–69	2467	1916	1790	93.4	2327	1722	1575	91.5	4794	3638	3365	92.5
70–79	1642	1231	1107	89.9	1382	1008	905	89.8	3024	2239	2012	89.9
80–84	462	243	197	81.1	368	228	172	75.4	830	471	369	78.3
Total	6828	4994	4551	91.1	6200	4259	3795	89.1	13,028	9253	8346	90.2

Values are numbers and proportions.

a Pre-marked sample consisting of a random sample of 20% aged 40–59 years and 50% aged 60–84 years (*n*=9925) as well as previous participants attending dual-energy X-ray absorptiometry, echocardiogram and/or eye examinations in Tromsø6 (*n*=3103).

b Invited when attending visit1.

c Of those attending visit1.

Study population characteristics including education level, mean levels or prevalences of cardiometabolic risk factors and conditions, and medication use are shown in Table III. The number of participants with measurements outside reference values is shown in Supplemental Table II. Altogether 98 participants were referred urgently to the UNN outpatient clinic due to abnormal blood pressure, oxygen saturation, potassium, creatine kinase or glucose levels. Adverse events were rare, of which fainting during examination was the most common (39 cases).

**Table III.** Descriptive characteristics of the participants by sex and age: the Tromsø Study 2015–2016.

	Women *N*=11,074					Men *N*=10,009				
	40–49 years *n*=3375	50–59 years *n*=3245	60–69 years *n*=2675	70–79 years *n*=1360	80–99 years *n*=412	40–49 years *n*=3053	50–59 years *n*=2788	60–69 years *n*=2502	70–79 years *n*=1315	80–99 years *n*=348
Body mass index, kg/m^2^	26.8 (5.2)	26.7 (4.9)	26.9 (4.7)	27.5 (5.0)	26.8 (4.5)	28.1 (4.2)	27.9 (4.0)	27.8 (3.9)	27.7 (3.9)	26.6 (3.5)
Waist circumference, cm	89.2 (13.3)	90.3 (12.7)	91.7 (12.5)	93.8 (12.9)	91.9 (11.8)	99.3 (11.8)	100.0 (11.0)	100.7 (11.2)	101.9 (10.9)	100.7 (10.0)
SBP, mmHg	116.6 (14.6)	123.0 (17.7)	132.5 (20.4)	143.5 (21.0)	152.2 (23.5)	126.7 (14.9)	130.9 (17.0)	135.8 (18.4)	140.4 (19.0)	144.4 (23.3)
DBP, mmHg	71.2 (9.3)	73.3 (9.7)	73.6 (9.7)	73.7 (9.8)	73.9 (10.7)	77.5 (9.4)	79.8 (9.8)	79.3 (9.6)	76.3 (9.7)	74.1 (10.2)
Total cholesterol, mmol/L	5.1 (0.9)	5.7 (1.0)	5.9 (1.1)	5.7 (1.1)	5.6 (1.1)	5.4 (1.0)	5.6 (1.0)	5.3 (1.1)	5.0 (1.1)	4.9 (1.1)
HDL-cholesterol, mmol/L	1.6 (0.4)	1.7 (0.5)	1.8 (0.5)	1.8 (0.5)	1.8 (0.5)	1.3 (0.4)	1.4 (0.4)	1.4 (0.4)	1.5 (0.4)	1.5 (0.4)
Triglycerides, mmol/L	1.2 (0.7)	1.4 (0.8)	1.5 (0.8)	1.4 (0.7)	1.4 (0.7)	1.9 (1.3)	1.8 (1.1)	1.6 (0.9)	1.5 (0.8)	1.3 (0.6)
HbA1c, %	5.4 (0.5)	5.7 (0.6)	5.8 (0.5)	5.9 (0.6)	6.0 (0.5)	5.5 (0.6)	5.7 (0.7)	5.8 (0.7)	5.9 (0.7)	6.0 (0.6)
Antihypertensives,^a^ %	7.0 (237)	15.0 (486)	30.2 (808)	53.8 (731)	66.7 (275)	8.3 (253)	18.4 (514)	39.4 (986)	55.5 (730)	62.1 (216)
LLD,^a^ %	1.8 (62)	7.6 (245)	20.0 (534)	34.3 (466)	32.3 (133)	4.5 (137)	11.9 (332)	25.1 (628)	39.9 (525)	39.7 (138)
MI,^a^ %	0.2 (6)	0.4 (12)	1.8 (45)	5.6 (71)	11.0 (40)	0.8 (25)	3.5 (94)	8.1 (197)	15.0 (191)	22.9 (72)
Stroke,^a^ %	0.7 (24)	1.3 (40)	2.5 (64)	4.4 (55)	9.1 (33)	0.8 (24)	1.9 (53)	4.4 (107)	8.1 (103)	13.7 (44)
Diabetes,^a^ %	2.6 (89)	4.4 (142)	7.1 (189)	11.7 (159)	12.1 (50)	3.6 (111)	6.1 (170)	10.3 (257)	13.9 (183)	16.4 (57)
Cancer,^a^ %	2.7 (89)	5.5 (173)	9.0 (232)	10.9 (138)	15.7 (58)	1.4 (43)	3.4 (93)	7.6 (183)	13.7 (176)	14.0 (46)
COPD,^a^ %	0.5 (18)	1.3 (41)	4.0 (102)	5.6 (70)	8.6 (31)	0.5 (14)	1.4 (38)	3.6 (88)	5.8 (73)	9.0 (29)
Obesity, %	23.0 (776)	21.1 (684)	21.9 (582)	26.8 (363)	21.9 (89)	26.9 (821)	25.2 (703)	24.7 (615)	23.8 (313)	15.2 (52)
Hypertension, %	13.9 (468)	28.2 (914)	51.4 (1374)	77.7 (1053)	89.5 (367)	24.9 (759)	41.1 (1144)	62.3 (1558)	77.8 (1021)	82.1 (285)
Hypercholesterolemia, %	5.8 (196)	20.0 (646)	35.9 (956)	47.0 (637)	46.0 (188)	14.2 (432)	23.5 (652)	34.1 (849)	44.9 (589)	45.5 (158)
Smoking,^a^ %	12.8 (430)	18.1 (581)	15.7 (416)	9.9 (132)	6.5 (26)	12.9 (392)	15.9 (4,41)	14.3 (355)	8.3 (108)	6.5 (22)
University education,^a^ %	68.7 (2303)	54.8 (1763)	40.3 (1061)	24.9 (323)	12.8 (47)	56.3 (1711)	47.1 (1299)	43.8 (1079)	38.3 (482)	26.7 (83)

Values are means (standard deviations) or proportions (numbers). Number varies (20,479–21,020) due to missing values.

a Self-reported.

SBP: systolic blood pressure; DBP: diastolic blood pressure; HbA1c: glycated haemoglobin; LLD: lipid-lowering drug; MI: myocardial infarction; COPD: chronic obstructive pulmonary disease; Obesity: body mass index ⩾30 kg/m^2^; Hypertension: blood pressure ⩾140/90 mmHg and/or self-reported use of antihypertensives; Hypercholesterolemia: total cholesterol ⩾7.0 mmol/L and/or low-density lipoprotein cholesterol ⩾5.0 mmol/L and/or self-reported use of LLDs; Smoking: current daily smoking.

## Discussion

### Attendance

Attendance was stable since Tromsø6 [29], believed to be due to broad marketing and outreach activity, and targeted recruitment efforts. We consider that continuous participant dialogue, strong community anchoring, and researcher involvement are key factors to maintain participation and development of ongoing population studies.

### New data collection methods and examinations

Extensive questionnaires may carry high participant burden. The introduction of online questionnaires to be completed before attendance (or on-site with technical support) contributed to high submission and completion. Online questionnaires reduced administrative burden, improved data quality with less risk of misclassification, and allowed for a hierarchal questionnaire structure with less participant burden. Digital illiteracy was a concern. However, telephone surveys by Statistics Norway indicate that digital literacy had increased, as 40% of 75–79-year-olds reported daily use of the internet in 2015, increasing to 70% in 2021 [30]. New questions about potentially sensitive topics and burdensome data collection were other concerns. However, we experienced high completion of all questions, and willingness to contribute to cumbersome data collection [9–11, 24].

### Risk factors and disease

Compared to previous surveys, there was a continued decrease in mean blood pressure [31], mean total cholesterol [32], smoking [33], and the prevalence of chronic obstructive pulmonary disease [34], and a continued increase in the prevalence of adiposity [35] and diabetes [36]. Despite the substantial increase in overweight and obesity, confirmed by body composition trends [37], the overall cardiovascular disease risk decreased [38].

## Conclusions

Tromsø7 participation was high despite the extensive data collection. The impact of population studies relies on study attendance, data quality and richness. We observed a continuous decline in cardiovascular risk factors whereas obesity continued to increase. The monitoring of risk factors and diseases is crucial to target public health interventions. New technology and strategies to improve data collection efficiency and decrease participant burden must be considered for future surveys. Nevertheless, traditional physical examinations and biological sampling are crucial, and interaction with study participants is key.

## Supplemental Material

sj-docx-1-sjp-10.1177_14034948221092294 – Supplemental material for The seventh survey of the Tromsø Study (Tromsø7) 2015–2016: study design, data collection, attendance, and prevalence of risk factors and disease in a multipurpose population-based health survey Click here for additional data file. Supplemental material, sj-docx-1-sjp-10.1177_14034948221092294 for The seventh survey of the Tromsø Study (Tromsø7) 2015–2016: study design, data collection, attendance, and prevalence of risk factors and disease in a multipurpose population-based health survey by Laila A. Hopstock, Sameline Grimsgaard, Heidi Johansen, Kristin Kanstad, Tom Wilsgaard and Anne Elise Eggen in Scandinavian Journal of Public Health

sj-docx-2-sjp-10.1177_14034948221092294 – Supplemental material for The seventh survey of the Tromsø Study (Tromsø7) 2015–2016: study design, data collection, attendance, and prevalence of risk factors and disease in a multipurpose population-based health survey Click here for additional data file. Supplemental material, sj-docx-2-sjp-10.1177_14034948221092294 for The seventh survey of the Tromsø Study (Tromsø7) 2015–2016: study design, data collection, attendance, and prevalence of risk factors and disease in a multipurpose population-based health survey by Laila A. Hopstock, Sameline Grimsgaard, Heidi Johansen, Kristin Kanstad, Tom Wilsgaard and Anne Elise Eggen in Scandinavian Journal of Public Health

sj-docx-3-sjp-10.1177_14034948221092294 – Supplemental material for The seventh survey of the Tromsø Study (Tromsø7) 2015–2016: study design, data collection, attendance, and prevalence of risk factors and disease in a multipurpose population-based health survey Click here for additional data file. Supplemental material, sj-docx-3-sjp-10.1177_14034948221092294 for The seventh survey of the Tromsø Study (Tromsø7) 2015–2016: study design, data collection, attendance, and prevalence of risk factors and disease in a multipurpose population-based health survey by Laila A. Hopstock, Sameline Grimsgaard, Heidi Johansen, Kristin Kanstad, Tom Wilsgaard and Anne Elise Eggen in Scandinavian Journal of Public Health

sj-docx-4-sjp-10.1177_14034948221092294 – Supplemental material for The seventh survey of the Tromsø Study (Tromsø7) 2015–2016: study design, data collection, attendance, and prevalence of risk factors and disease in a multipurpose population-based health survey Click here for additional data file. Supplemental material, sj-docx-4-sjp-10.1177_14034948221092294 for The seventh survey of the Tromsø Study (Tromsø7) 2015–2016: study design, data collection, attendance, and prevalence of risk factors and disease in a multipurpose population-based health survey by Laila A. Hopstock, Sameline Grimsgaard, Heidi Johansen, Kristin Kanstad, Tom Wilsgaard and Anne Elise Eggen in Scandinavian Journal of Public Health

sj-docx-5-sjp-10.1177_14034948221092294 – Supplemental material for The seventh survey of the Tromsø Study (Tromsø7) 2015–2016: study design, data collection, attendance, and prevalence of risk factors and disease in a multipurpose population-based health survey Click here for additional data file. Supplemental material, sj-docx-5-sjp-10.1177_14034948221092294 for The seventh survey of the Tromsø Study (Tromsø7) 2015–2016: study design, data collection, attendance, and prevalence of risk factors and disease in a multipurpose population-based health survey by Laila A. Hopstock, Sameline Grimsgaard, Heidi Johansen, Kristin Kanstad, Tom Wilsgaard and Anne Elise Eggen in Scandinavian Journal of Public Health
