# Supplementary material for: The seventh survey of the Tromsø Study (Tromsø7) 2015–2016: study design, data collection, attendance, and prevalence of risk factors and disease in a multipurpose population-based health survey
Source: Scand J Public Health. 2022 May 4;50(7):919–29. doi: 10.1177/14034948221092294 (PMC9578102; doi:10.1177/14034948221092294)
Supplement: sj-docx-1-sjp-10.1177_14034948221092294 – Supplemental material for The seventh survey of the Tromsø Study (Tromsø7) 2015–2016: study design, data collection, attendance, and prevalence of risk factors and disease in a multipurpose population-based health survey [file sj-docx-1-sjp-10.1177_14034948221092294.docx]

**Supplementary Table 1.** An overview of biological sample analyses’ methods. The Tromsø Study 2015-2016.

| Material | Analysis | N | Instrument | Method | CVA |
| --- | --- | --- | --- | --- | --- |
| Visit1 | | | | | |
| Serum | Sodium | 20,992 | Cobas 8000/Roche | Ion-selective electrode, indir. | 0.8 % at 141 mmol/L |
| Serum | Potassium | 20,962 | Cobas 8000/Roche | Ion-selective electrode, indir. | 0.9 % at 4.2 mmol/L |
| Serum | Chloride | 20,992 | Cobas 8000/Roche | Ion-selective electrode, indir. | 0.9 % at 102 mmol/L |
| Serum | Calcium | 3,517 | Cobas 8000/Roche | Colorimetric | 1.2 % at 2.32 mmol/L |
| Serum | Albumin | 3,517 | Cobas 8000/Roche | Colorimetric endpoint | 1.6 % at 43 g/L |
| Serum | Creatinine | 20,990 | Cobas 6000/Roche | Enzymatic colorimetric | 1.75 % at 73 µmol/L |
| Serum | CK | 20,964 | Cobas 8000/Roche | Photometric | 1.5 % at 134 U/L |
| Serum | hsCRP | 20,972 | Cobas 8000/Roche | Immunturbidimetric | 3.2 % at 0.7 mg/L |
| Serum | T-C | 20,972 | Cobas 8000/Roche | Enzymatic colorimetric | 1.4 % at 5.0 mmol/L |
| Serum | LDL-C | 20,971 | Cobas 8000/Roche | Hom. enzymatic colorimetric | 1.5 % at 3.0 mmol/L |
| Serum | HDL-C | 20,972 | Cobas 8000/Roche | Hom. enzymatic colorimetric | 1.6 % at 1.5 mmol/L |
| Serum | Triglycerides | 20,972 | Cobas 8000/Roche | Colorimetric | 1.7 % at 1.4 mmol/L |
| Serum | Glucose | 20,992 | Cobas 8000/Roche | UV hexokinase | 1.0 % at 5.4 mmol/L |
| Serum | Vitamin D2 | 20,922 | Xevo TQ-S/Waters | LC-MS/MS | 12.4 % at 97.3 nmol/L |
| Serum | Vitamin D3 | 20,922 | Xevo TQ-S/Waters | LC-MS/MS | 8.2 % at 96.3 nmol/L |
| Plasma | PTH | 3,769 | Cobas 6000/Roche | ECLIA, sandwich | 3.9 % at 4.1 pmol/L |
| Blood | HbA1c | 20,813 | Tosoh G8/Alere | HPLC | 0.87 % at 5.08 % |
| Blood | Hemoglobin | 20,868 | Sysmex XN/Sysmex | Photometri | <3 % |
| Blood | Hematokrit | 20,865 | Sysmex XN/Sysmex | Impedance, hydro. focusing | <2 % |
| Blood | Trombocytes | 20,844 | Sysmex XN/Sysmex | Impedance, hydro. focusing | <6 % |
| Blood | RBC | 20,864 | Sysmex XN/Sysmex | Impedance, hydro. focusing | <2 % |
| Blood | WBC | 20,869 | Sysmex XN/Sysmex | FFC with half led laser | <4 % |
| Blood | MCHC | 20,865 | Sysmex XN/Sysmex | Estimated value | <2 % |
| Blood | MCH | 20,865 | Sysmex XN/Sysmex | Estimated value | <2 % |
| Blood | RDW-CV | 20,864 | Sysmex XN/Sysmex | Estimated value | <3 % |
| Blood | MPV | 20,832 | Sysmex XN/Sysmex | Estimated value | <2 % |
| Blood | MCV | 20,865 | Sysmex XN/Sysmex | Estimated value | <2 % |
| Visit2 | | | | | |
| Serum | Sodium | 8,330 | Cobas 8000/Roche | Ion-selective electrode, indir. | 0.8 % at 141 mmol/L |
| Serum | Potassium | 8,312 | Cobas 8000/Roche | Ion-selective electrode, indir. | 0.9 % at 4.2 mmol/L |
| Serum | Chloride | 8,330 | Cobas 8000/Roche | Ion-selective electrode, indir. | 0.9 % at 102 mmol/L |
| Serum | Glucose | 8,330 | Cobas 8000/Roche | UV hexokinase | 1.0 % at 5.4 mmol/L |
| Serum | Creatinine | 8,328 | Cobas 6000/Roche | Enzymatic colorimetric | 1.75 % at 73 µmol/L |
| Serum | Cystatin C | 8,330 | Cobas 8000/Roche | Immunturbidimetric | 3.2 % at 0.78 mg/L |
| Serum | Uric acid | 8,330 | Cobas 8000/Roche | Enzymatic colorimetric | 1.2 % at 287 µmol/L |
| Serum | hsCRP | 2,089 | Cobas 8000/Roche | Immunturbidimetric | 3.2 % at 0.78 mg/L |
| Serum | NT-proBNP | 2,115 | Cobas 8000/Roche | ECLIA, sandwich | 3.6 % at 195 pg/mL |
| Serum | Troponin-T | 2,125 | Cobas 8000/Roche | ECLIA, sandwich | 4.4 % at 18 ng/L |
| Blood | T WBC diff c | 7,349 | Sysmex XN/Sysmex | FFC | <4 % |
| Urine | Sodium | 7,821 | Cobas 8000/Roche | Ion-selective electrode, indir. | 0.8 % at 141 mmol/L |
| Urine | Potassium | 7,821 | Cobas 8000/Roche | Ion-selective electrode, indir. | 1.6 % at 31 mmol/L |
| Urine | Chloride | 7,821 | Cobas 8000/Roche | Ion-selective electrode, indir. | 2.2 % at 81 mmol/L |
| Urine | Albumin | 7,821 | Cobas 8000/Roche | Colorimetric endpoint | 2.6 % at 62 mg/L |
| Urine | Creatinine | 7,821 | Cobas 8000/Roche | Enzymatic colorimetric | 2.3 % at 5.7 mmol/L |
| Urine | Urea | 7,821 | Cobas 8000/Roche | Kinetic UV | 2.5 % at 262 mmol/L |
| Urine | NAG | 7,821 | Cobas 8000/Roche | Colorimetric | 2.2 % at 43 U/L |
| Additional material stored for analysis of environmental pollutants, RNA, DNA, etc. and for the biobank | | | | | |

N is number of participants (for urine; 1^st^ day).

CVA, coefficient of variation for analytical variability; CK, creatine kinase; hsCRP, high-sensitive C-reactive protein; T, total; C, cholesterol; LDL, low density lipoprotein; HDL, high-density lipoprotein; PTH, parathyroid hormone; HbA1c, glycated hemoglobin; RBC, red blood cell; WBC, white blood cell; MCHC, mean corpuscular hemoglobin concentrate; MCH, mean corpuluscular haemoglobin; RDW-CV, red blood cell distribution width; MPV, mean platelet volume; MCV, mean cell volume; NT-proBNP, N-terminal pro-b-type natriuretic peptide; Diff, differential; c, count; NAG; N-acetyl-β-D-glucosaminidase; indir., indirect; hom, homogen; UV, ultra violet; LC, liquid chromatography; MS, mass spectrometry; HPLC, high-performance liquid chromatography; ECLIA, electrochemiluminescense immunoassay; hydro., hydrodynamic; FFC, fluorescence flow cytometry.
