# Supplementary material for: The seventh survey of the Tromsø Study (Tromsø7) 2015–2016: study design, data collection, attendance, and prevalence of risk factors and disease in a multipurpose population-based health survey
Source: Scand J Public Health. 2022 May 4;50(7):919–29. doi: 10.1177/14034948221092294 (PMC9578102; doi:10.1177/14034948221092294)
Supplement: sj-docx-2-sjp-10.1177_14034948221092294 – Supplemental material for The seventh survey of the Tromsø Study (Tromsø7) 2015–2016: study design, data collection, attendance, and prevalence of risk factors and disease in a multipurpose population-based health survey [file sj-docx-2-sjp-10.1177_14034948221092294.docx]

**Supplementary Table 2.** Feedback system for deviant test results. The Tromsø Study 2015-2016.

| Examination | | N | Immediate action  Cut-off | Action | n | Letter  Cut-off | Letter recommendation | **n** |
| --- | --- | --- | --- | --- | --- | --- | --- | --- |
| SBP^1^ | | 21,008 | ≥220 mmHg | Individual at-side action | 16 | ≥160 mmHg | *“The result may indicate high blood pressure. If you are not already followed-up by your GP for blood pressure, control of the blood pressure by the GP within approximately 1 month is recommended.”* | 1,562 |
| DBP^1^ | | 21,008 | ≥120 mmHg | Individual at-side action | 5 | ≥100 mmHg | *“The result may indicate high blood pressure. If you are not already followed-up by your GP for blood pressure, control of the blood pressure by the GP within approximately 1 month is recommended.”* | 282 |
| SpO_2_ | | 21,053 | ≤92 % | Individual at-side action | 58 | <70 years: 94 %  ≥70 years: 93 % | *“Your oxygen saturation was measured to be X%, which is lower than normal. By examining the bloodstream in a fingertip, we have measured to what extent the red blood cells are saturated with oxygen after your blood has passed through your lungs. Oxygen saturation is normally 96-100%. Conditions that may give low oxygen saturation are: High age, lung disease, heart disease, smoking and significant obesity. If you are more short-winded than others in your age and you are not already being treated for heart- or lung disease, you can consider contact your GP. This applies in particular if your oxygen saturation is 92% or lower. The GP will not be sent your results, so bring this letter to the GP. If you are invited to Visit2 of the Tromsø Study, your oxygen saturation will be controlled again, and you do not need to go to the GP before this has been performed.”* | 247 |
| Sodium | | 20,992 | <125 mmol/L or >155 mmol/L | Telephone contact | 0 | None | NA | 0 |
| Potassium | | 20,962 | <2.5 mmol/L or >6.0 mmol/L | Telephone contact | 1 | 2.5-2.9 mmol/L | *“In the Tromsø Study, potassium was measured in your blood. You had a value of X g/L, which shows that you have too low level of potassium.* *Unless you are already under screening or treatment, we recommend that you have this controlled by your GP within the next week. When you visit the GP, you should bring this letter so that the CP can see the result.”* | 1 |
|  |  |  |  |  |  | 5.5-6.0 mmol/L | *“In the Tromsø Study, potassium was measured in your blood. You had a value of X g/L, which shows that you have too high level of potassium.* *Unless you are already under screening or treatment, we recommend that you have this controlled by your GP within the next week. When you visit the GP, you should bring this letter so that the CP can see the result.”* | 15 |
| Calcium | 3,517 | | <1.8 mmol/L or >3.0 mmol/L | Telephone contact | 0 | 1.8-<2.0 mmol/L | *“In the Tromsø Study, the calcium level was measured in your blood. You had a value of X mmol/L, which may indicate that you have too low level of potassium. You will be offered follow-up at the Medical department at the University Hospital of North Norway. The GP does not have access to your results. If you visit your GP, you should bring this letter so that the GP is informed.”* | 1 |
|  |  |  |  |  |  | 2.65-2.85 mmol/L | *“In the Tromsø Study, the calcium level was measured in your blood. You had a value of X mmol/L, which may indicate that you have too high level of potassium. If this is new to you, we recommend you to contact your GP for further follow-up. The GP does not have access to your results. When you visit your GP, you should bring this letter so that the GP can see the result.”* | 35 |
|  |  |  |  |  |  | >2.85-3.0 mmol/L | *“In the Tromsø Study, the calcium level was measured in your blood. You had a value of X mmol/L, which may indicate that you have too high level of potassium. You will be offered follow-up at the Medical department at the University Hospital of North Norway. The GP does not have access to your results. If you visit your GP, you should bring this letter so that the GP is informed.”* | 2 |
| Albumin | | 3,517 | <25 g/L | Telephone contact | 0 | 25 g/L-30 g/L | *“In the Tromsø Study, albumin was measured in your blood. You had a value of X g/L. This may indicate too low level of albumin.* *Unless you are already under screening or treatment, we recommend that you have this controlled by your GP within the next week. The GP does not have access to your results. When you visit the GP, you should bring this letter.”* | 0 |
| Creatinine | | 20,990 | >400 µmol/L | Telephone contact | 0 | ≥75 years:  W: >135 µmol/L  M: >175 µmol/L  <75 years:  W: >90 µmol/L  M: >105 µmol/L | *“In the Tromsø Study, the creatinine was measured in the blood, and you had the value of X μmol/L. The analysis of creatinine in the blood shows a too high level. This may indicate that your kidneys have reduced capacity, that is, you may have kidney impairment. If this is new to you, we recommend that you contact your GP for control. If you are not already under screening or treatment, we recommend that you have this controlled by your own GP. The GP does not have access to your results. When you visit the GP, you should bring this letter so that the doctor can see the result.”* | 644 |
| CK | | 20,964 | ≥5000U/L | Telephone contact | 4 | W: >630 U/L  M <50 years: >1200 U/L  M ≥50 years: >840 U/L | *“In the Tromsø Study, creatin kinase was measured from your blood. You had a value of X mmol/L, which may indicate that you have too high level of creatin kinase. This can indicate affection of muscles but does not need to indicate serious disease. We therefore recommend you to take a new test at the University Hospital of North Norway. Attached you will find a requisition that you bring with you when you contact the Blood taking unit to take the test.* [information about location, opening hours, waiting time and preparations]. *If both of your test shows elevated values, you will be referred to further follow-up at the Neurology department. If you do not hear anything after the second test, this means that you do not need referral for further follow-up.”* | 93 |
| Uric acid | | 8,330 | None | NA | NA | W: >600 µmol/  M: >700 µmol/L | *“In the Tromsø Study, the level of uric acid was measured in your blood. You had a value of X μmol/L. This shows that the level of uric acid in your blood is too high. Unless you are already under screening or treatment, we recommend that you have this controlled by your GP. The GP does not have access to your results. When you visit the GP, you should bring this letter.”* | 7 |
| hsCRP | | 20,972 | None | NA | NA | ≥25 mg/L | *“C-reactive protein (CRP) is a protein produced in the body in response to inflammatory conditions. Significantly elevated values are measured in acute infections, chronic inflammatory conditions, and other serious diseases. In the Tromsø Study your CRP value was X mg/L. If you were not acutely ill when you attended the Tromsø Study and do not have a chronic inflammatory condition with elevated CRP-values, you should have this controlled by your GP within 2-3 weeks. The GP does not have access to your results. When you visit the GP, you should bring this letter, so that the CP can see the result.”* | 113 |
| T-C^1^ | | 20,972 | None | NA | NA | ≥8 mmol/L | *“The result may indicate disturbances in the lipid metabolism, high cholesterol. If you are not already followed-up by your GP for this, control by the GP within approximately 3 months is recommended.”* | 299 |
| Glucose | | 20,992 | No diabetes^2^  >20 mmol/L  Diabetes^2^  >25 mmol/L | Telephone contact | 14 | None | NA | NA |
| HbA1C^1^ | | 20,813 | None | NA | NA | ≥6.5 % | *“Your long-term level of blood sugar shows a too high value, and it may indicate that you have diabetes. If this is new to you, we recommend you to contact your GP within approximately 1 month for further follow-up.”* | 1,086 |
|  |  |  |  |  |  | 6.1-6.4 % | *“Your long-term level of blood sugar shows a value in the borderline between what is normal and what diabetes. We recommend that you have this controlled by your GP when suitable and at the latest within 1 year.”* | 1,399 |
| Hemoglobin^1^ | | 20,868 | <8 g/dl | Telephone contact | 0 | W: <9 g/dl  M: <10 g/dl | *“You have a hemoglobin value (blood percentage) that may indicate to low hemoglobin level. If this is new to you, we recommend you to contact your GP within approximately 1 month for a new test and further follow-up.”* | 14 |
| Thrombocytes | | 20,844 | <15x10^9^/L | Telephone contact | 0 | 15-<50x10^9^/L | *“In the Tromsø Study, the level of hemoglobin (blood percentage), white blood cells and blood platelets were measured in your blood. You had a value for blood platelets of X 10^9^/L. This indicates that you have too low level of blood platelets. If this is new to you, we recommend that you contact your GP within 1 week after attending the Tromsø Study dd.mm.yy, for a new test and further follow-up. The GP does not have access to your results. When you visit the GP, you should bring this letter, so that the CP can see the result.”* | 4 |
|  |  |  |  |  |  | 50-<80x10^9^/L | *“In the Tromsø Study, the level of hemoglobin (blood percentage), white blood cells and blood platelets were measured in your blood. You had a value for blood platelets of X 109/L. This indicates that you have too low level of blood platelets. If this is new to you, we recommend you to contact your GP within 1 month after attending the Tromsø Study dd.mm.yy, for a new test and further follow-up. The GP does not have access to your results. When you visit your GP, you should bring this letter so that the GP can see the result.”* | 9 |
|  |  |  |  |  |  | >600x10^9^/L | *“In the Tromsø Study, the level of hemoglobin (blood percentage), white blood cells and blood platelets were measured in your blood. You had a value for blood platelets of X 109/L. This indicates that you have too high level of blood platelets. If this is new to you, we recommend you to contact your GP within 1 month after attending the Tromsø Study dd.mm.yy, for a new test and further follow-up. The GP does not have access to your results. When you visit your GP, you should bring this letter so that the GP can see the result.”* | 19 |
| WBC | | 20,869 | <1x10^9^/L | Telephone contact | 0 | <2.5x10^9^/L | *“In the Tromsø Study, the level of hemoglobin (blood percentage), white blood cells and blood platelets were measured in your blood. You had a value for white blood cells of X 10^9^/L. This indicates that you have too low levels of white blood cells. If this is new to you, we recommend to contact your GP within approximately 1 month after attending the Tromsø Study dd.mm.yy, for a new test and further follow-up. The GP does not have access to your results. When you visit the GP, you should bring this letter, so that the CP can see the result.”* | 7 |
|  |  |  |  |  |  | ≥20x10^9^/L | *“In the Tromsø Study, the level of hemoglobin (blood percentage), white blood cells and blood platelets were measured in your blood. You had a value for white blood cells of X 109/L. This indicates that you have too high levels of white blood cells. If this is new to you, we recommend to contact your GP within approximately 1 month after attending the Tromsø Study dd.mm.yy, for a new test and further follow-up. The GP does not have access to your results. When you visit the GP, you should bring this letter, so that the CP can see the result.”* | 12 |
| Anti-HCV | | 20,946 | None | NA | NA | Reactive | *In the Tromsø Study, you took a blood test. The test show that you have antibodies against hepatitis C which means that you have or have had a hepatitis C infection. We will ensure that you will be offered follow-up from the University Hospital of North Norway (UNN) and that a consultant at the Gastromedical department will contact you.* | 217 |
| ACR | | 3,517 | None | NA | NA | >34 mg/mmol (median from the 3 samples) | *“The results from analysis of creatinine and albumin show an albumin-creatinine ratio of X mg/mmol, which means protein in the urine. If this is new to you, we recommend you to visit your GP for control. The GP does not have access to your results. When you visit the GP, you should bring this letter so that the GP can see the result.”* | 40 |

N is number of examined participants. n is number of participants with deviant test results.

^1^Results from Visit1 given in standard letter to all participants together with height, weight, body mass index, and high-density lipoprotein cholesterol including information about each measurement with reference values, and a general feedback (*“In this letter you will have results from some of the measurements performed at the Tromsø Study. Some results can lead to us recommending follow-up at the GP. This will be found under “recommendations”. The GP does not have access to your results. The GP will not be sent your results. Therefore, bring this letter with you if you visit your GP due to your recommendations or your own questions about the results.”*).

^2^Self-reported.

SBP, systolic blood pressure; DBP, diastolic blood pressure; SpO2, oxygen saturation; CK, creatine kinase; hsCRP, high-sensitive C-reactive protein; T, total; C, cholesterol; HbA1c, glycated hemoglobin; WBC, white blood cell; Anti-HCV, hepatitis C virus antibodies; ACR, albumin-creatinine ratio; W, women; M, men; GP, general practitioner.
