# Supplementary material for: The seventh survey of the Tromsø Study (Tromsø7) 2015–2016: study design, data collection, attendance, and prevalence of risk factors and disease in a multipurpose population-based health survey
Source: Scand J Public Health. 2022 May 4;50(7):919–29. doi: 10.1177/14034948221092294 (PMC9578102; doi:10.1177/14034948221092294)
Supplement: sj-docx-3-sjp-10.1177_14034948221092294 – Supplemental material for The seventh survey of the Tromsø Study (Tromsø7) 2015–2016: study design, data collection, attendance, and prevalence of risk factors and disease in a multipurpose population-based health survey [file sj-docx-3-sjp-10.1177_14034948221092294.docx]

**Supplementary Table 3.** Attendance at Visit1 according to sex, age, and previous attendance. The Tromsø Study 2015-2016.

|  | Women | | | | Men | | | |
| --- | --- | --- | --- | --- | --- | --- | --- | --- |
|  | First time invitees | | Previously attended | | First time invitees | | Previously attended | |
| Age, years | Invited | % | Invited | % | Invited | % | Invited | % |
| 40-49 | 3,403 | 59.6 | 1,792 | 75.3 | 3,995 | 48.3 | 1,567 | 71.7 |
| 50-59 | 1,044 | 47.7 | 3,490 | 78.7 | 1,293 | 41.2 | 3,034 | 74.4 |
| 60-69 | 380 | 45.8 | 3,206 | 78.1 | 490 | 38.8 | 3,053 | 75.7 |
| 70-79 | 92 | 29.3 | 1,909 | 69.9 | 94 | 26.6 | 1,803 | 71.5 |
| 80-89 | 46 | 21.7 | 935 | 40.5 | 17 | 29.4 | 622 | 51.4 |
| 90-104 | 16 | 0.0 | 226 | 10.6 | 6 | 0.0 | 78 | 29.5 |
| Total | 4,981 | 55.0 | 11,558 | 72.1 | 5,895 | 45.5 | 10,157 | 72.1 |

Values are numbers and proportions.
