# Supplementary material for: The seventh survey of the Tromsø Study (Tromsø7) 2015–2016: study design, data collection, attendance, and prevalence of risk factors and disease in a multipurpose population-based health survey
Source: Scand J Public Health. 2022 May 4;50(7):919–29. doi: 10.1177/14034948221092294 (PMC9578102; doi:10.1177/14034948221092294)
Supplement: sj-docx-4-sjp-10.1177_14034948221092294 – Supplemental material for The seventh survey of the Tromsø Study (Tromsø7) 2015–2016: study design, data collection, attendance, and prevalence of risk factors and disease in a multipurpose population-based health survey [file sj-docx-4-sjp-10.1177_14034948221092294.docx]

**Supplementary Table 4.** Attendance according to country of birth. The Tromsø Study 2015-2016.

| Country/Region | Invited | Attended | % |
| --- | --- | --- | --- |
| Norway | 29,730 | 19,818 | 66.7 |
| Western Countries^1^ | 1,320 | 743 | 56.3 |
| Eastern Europe^2^ | 752 | 196 | 26.1 |
| Other Countries^3^ | 789 | 326 | 41.3 |
| Total | 32,591 | 21,083 | 64.7 |

Values are numbers and proportions. Information about country of birth is from the National Population Register.

^1^Western Europe, North America, Oceania.

^2^Eastern Europe including Russia.

^3^Asia, Africa, South America
