# Supplementary material for: The seventh survey of the Tromsø Study (Tromsø7) 2015–2016: study design, data collection, attendance, and prevalence of risk factors and disease in a multipurpose population-based health survey
Source: Scand J Public Health. 2022 May 4;50(7):919–29. doi: 10.1177/14034948221092294 (PMC9578102; doi:10.1177/14034948221092294)
Supplement: sj-docx-5-sjp-10.1177_14034948221092294 – Supplemental material for The seventh survey of the Tromsø Study (Tromsø7) 2015–2016: study design, data collection, attendance, and prevalence of risk factors and disease in a multipurpose population-based health survey [file sj-docx-5-sjp-10.1177_14034948221092294.docx]

**Supplementary Table 5.** Attendance at Visit2 according to sex, age, and whether the invitees were randomly selected to Visit2. The Tromsø Study 2015-2016.

|  | Random | | | | Not random^1^ | | | |
| --- | --- | --- | --- | --- | --- | --- | --- | --- |
| Age, years | Eligible | Invited | Attended | % | Eligible | Invited | Attended | % |
| Women |  |  |  |  |  |  |  |  |
| 40-49 | 1,047 | 691 | 621 | 89.9 | 24 | 22 | 20 | 90.9 |
| 50-59 | 928 | 652 | 590 | 90.5 | 258 | 239 | 226 | 94.6 |
| 60-69 | 1,821 | 1,342 | 1,250 | 93.1 | 646 | 574 | 540 | 94.1 |
| 70-79 | 992 | 677 | 601 | 88.8 | 650 | 554 | 506 | 91.3 |
| 80-84 | 276 | 128 | 105 | 82.0 | 186 | 115 | 92 | 80.0 |
| Total | 5,064 | 3,490 | 3,167 | 90.7 | 1,764 | 1,504 | 1,384 | 92.0 |
| Men |  |  |  |  |  |  |  |  |
| 40-49 | 1,105 | 607 | 529 | 87.1 | 12 | 11 | 11 | 100.0 |
| 50-59 | 835 | 536 | 463 | 86.4 | 171 | 147 | 140 | 95.2 |
| 60-69 | 1,743 | 1,217 | 1,103 | 90.6 | 584 | 505 | 472 | 93.5 |
| 70-79 | 963 | 660 | 587 | 88.9 | 419 | 348 | 318 | 91.4 |
| 80-84 | 215 | 129 | 94 | 72.9 | 153 | 99 | 78 | 78.8 |
| Total | 4,861 | 3,149 | 2,776 | 88.2 | 1,339 | 1,110 | 1,019 | 91.8 |

Values are numbers and proportions.

^1^Participation at dual energy X-ray-, echocardiogram- and/or eye examinations in Tromsø6.
